# Supplementary material for: The interactome of intact mitochondria by cross-linking mass spectrometry provides evidence for coexisting respiratory supercomplexes
Source: Mol Cell Proteomics. 2017 Dec 8;17(2):216–32. doi: 10.1074/mcp.RA117.000470 (PMC5795388; doi:10.1074/mcp.RA117.000470)
Supplement: Supplemental Data [file supp_RA117.000470_133922_0_supp_23978_fzffwf.zip › spectra_annotation/mito_spectra_annotation/225-1-2-1-9-1.pdf]

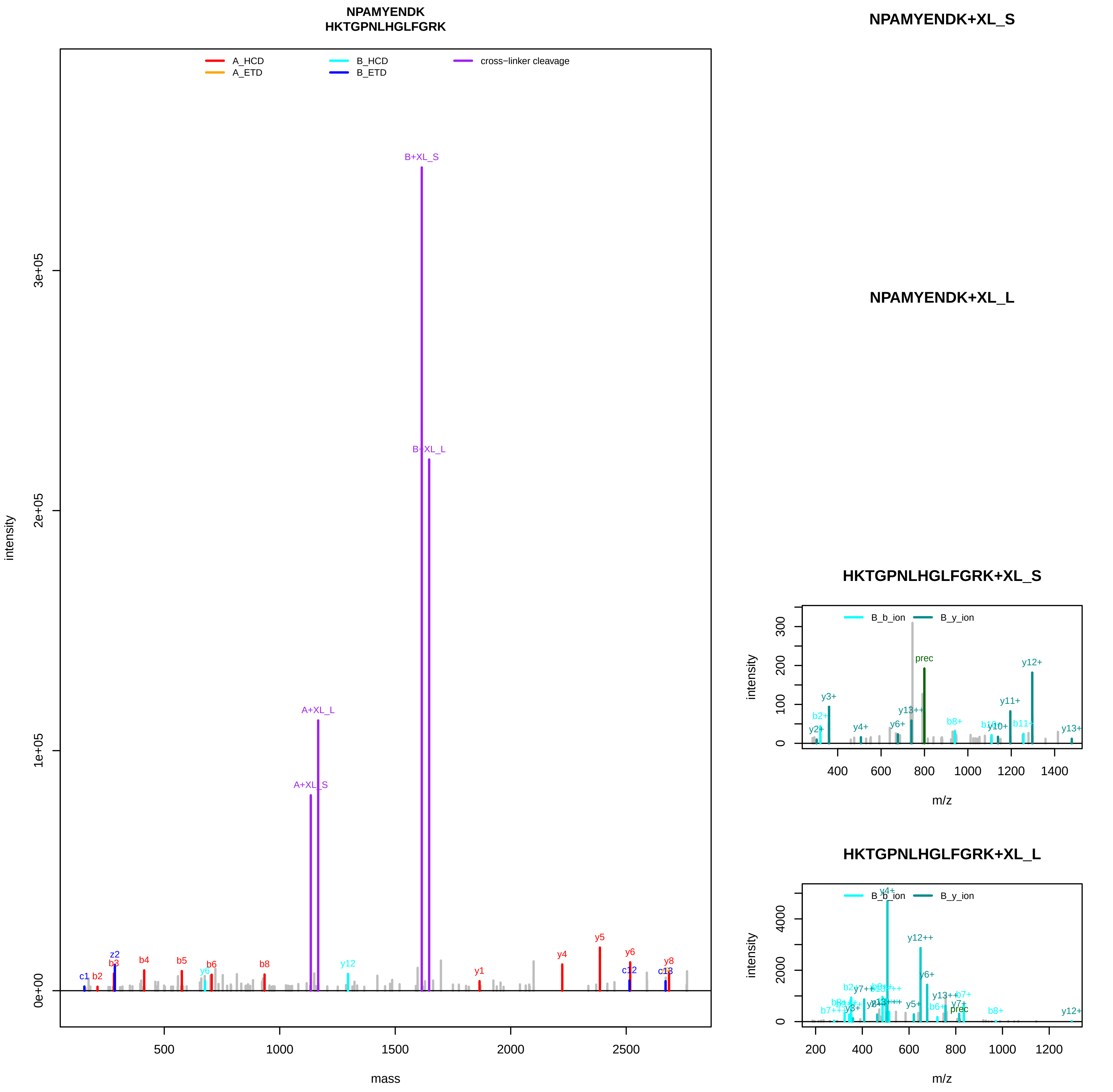
NPAMYENDK
HKTGPNLHGLFGRK
A_HCD
A_ETD
B_HCD
B_ETD
cross−linker cleavage
B+XL_S
B+XL_L
A+XL_L
A+XL_S
intensity
mass
0e+00
1e+05
2e+05
3e+05
500
1000
1500
2000
2500
c1
b2
z2
b3
b4
b5
y6
b6
b8
y12
y1
y4
y5
y6
c12
c13
y8
NPAMYENDK+XL_S
NPAMYENDK+XL_L
HKTGPNLHGLFGRK+XL_S
B_b_ion
B_y_ion
prec
y12+
y3+
y13++
y11+
b2+
y2+
y4+
y6+
b8+
y10+
b11+
y13+
m/z
HKTGPNLHGLFGRK+XL_L
y4+
y12++
y6+
y13++
b7+
y7+
prec
y5+
b6+
b8+
y12+
y8+
